# Inhibitory effects of Kratom constituents, mitragynine and 7-hydroxymitragynine, on 4-methylumbelliferone glucuronidation by human UDP-glucuronosyltransferases

**DOI:** 10.1016/j.toxrep.2025.101951

**Published:** 2025-02-10

**Authors:** Verawan Uchaipichat

**Affiliations:** Division of Clinical Pharmacy, Faculty of Pharmaceutical Sciences, Khon Kaen University, Khon Kaen, Thailand

**Keywords:** Glucuronidation, Herb-Drug Interaction, 7-Hydroxymitragynine, Kratom, Mitragynine, UDP-glucuronosyltransferases

## Abstract

As Kratom use increases, concerns about potential herb-drug interactions with liver enzymes, particularly UDP-glucuronosyltransferases (UGTs), have emerged. This study investigated the inhibitory effects of Kratom leaf constituents, mitragynine and 7-hydroxymitragynine, on 4-methylumbelliferone (4MU) glucuronidation by a panel of recombinant human UGT enzymes, including UGT1A1, UGT1A3, UGT1A6, UGT1A9, UGT2B7, and UGT2B15. The degree of inhibition exhibited by mitragynine and 7-hydroxymitragynine on UGTs varied. Mitragynine exhibited the highest inhibitory potency on UGT1A3 with an IC_50_ value of 72 µM. Moderate inhibition potency of mitragynine were observed for UGT1A6, UGT1A9 and UGT2B15, with IC_50_ value of 121, 131, and 152 µM, respectively, whereas the inhibition on UGT1A1 and UGT2B7 was low (IC_50_ > 200 µM). 7-Hydroxymitragynine exhibited the highest inhibitory potency on UGT1A9, with IC_50_ value of 51 µM, while moderate potency was observed for UGT1A1 and UGT1A3, with IC_50_ value of 196 and 141 µM, respectively. The inhibitory potency of 7-hydroxymitragynine on UGT2B15 was low (IC_50_ > 200 µM), while negligible effects were observed for UGT1A6 and UGT2B7. Kinetic inhibition study revealed that mitragynine noncompetitively inhibited UGT1A3 (K_i_ = 45 µM) and competitively inhibited UGT1A9 (K_i_ = 114 µM), while 7-hydroxymitragynine competitively inhibited UGT1A3 (K_i_ = 33 µM) and noncompetitively inhibited UGT1A9 (K_i_ = 29 µM). The experimental K_i_ values found here are relatively high compared to the maximum plasma concentrations of mitragynine and 7-hydroxymitragynine reported in humans, suggesting an unlikely potential for herb-drug interactions via UGT inhibition.

## Highlights

• As Kratom use rises, concerns grow about its potential herb-drug interactions with UGTs.
• The inhibition degree by mitragynine and 7-hydroxymitragynine on UGTs varied.
• Mitragynine showed potential *in vitro* inhibition on UGT1A3, and 7-hydroxymitragynine on UGT1A9.

## Graphical Abstract

**Figure:**
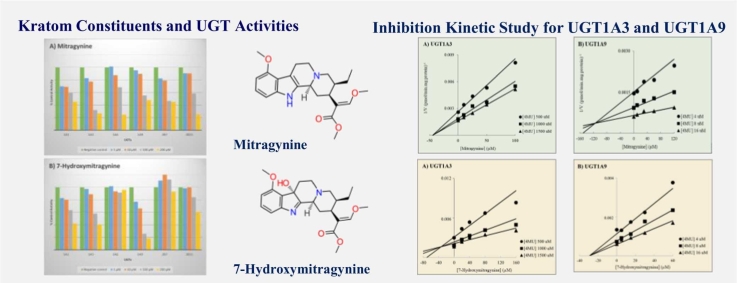

## 1 Introduction

*Mitragyna speciosa*, known as “Kratom” in Thailand, is a tropical plant mainly found in Southeast Asian countries, particularly in Thailand and Malaysia [1]. Traditionally, Kratom leaves have been used for medicinal purposes such as antitussive, antidiarrheal, analgesic and antidiabetic, and recreational purposes [1], [2]. Among these, antinociceptive and psychoactive properties of Kratom has been investigated extensively [3], [4]. Kratom’s leaves contain many alkaloids. Among the alkaloid content in the extract, mitragynine is the major compound, comprising 66 %, followed by paynantheine (9 %), speciogynine (7 %), 7-hydroxymitragynine (2 %), speciociliatine (1 %), and other alkaloids with contents of less than 1 % such as speciophylline and mitraphylline [5], [6]. Of these, mitragynine and 7-hydroxymitragynine pose the opioid-like effects, where the latter is more potent analgesic than mitragynine and morphine [3], [4], [5], [6]. Due to its biological effects, Kratom has been used in some countries for medical purposes to treat pain and opioid withdrawal symptoms [5]. In Thailand, particularly in the southern region, Kratom leaves have traditionally been used for their stimulant and analgesic properties, primarily by laborers to combat fatigue and enhance productivity [5], [7]. Additionally, recreational use of Kratom has gained popularity among younger populations, often in the form of mixtures such as "4 × 100", a cocktail containing Kratom, cough syrup, soda, and other substances [5], [7]. However, Kratom consumption can lead to addiction [3], [5], [6]; thus, the legal status of Kratom varies between countries. After being lawfully prohibited since 1943, Kratom was recently removed from the Schedule 5 drugs under the Thai Narcotics Act and can now be legally used in Thailand. Owing to the unlock scheme and more convenient access, the widespread use of Kratom may potentiate the prevalence of herb-drug interaction.

The common mechanism underlying herb-drug interactions involves the inhibition of drug-metabolizing enzymes, such as cytochrome P450s (CYPs) and UDP-glucuronosyltransferases (UGTs), by constituent compounds in herbs [8]. Regarding Kratom, the inhibitory effects of Kratom extract and its major biologically active constituents on various CYP isoforms have been extensively studied [9], with the most potent inhibition observed on CYP2D6 and CYP3A activities [10], [11], [12], [13], [14], [15]. Of note, suspected Kratom-drug interactions arising from the inhibition of CYP3A, CYP2D6, and/or P-glycoprotein have been reported in several fatal cases [9]. However, the systematic investigation for the inhibition effects of Kratom’s constituents on the activities of UGTs has been limited.

UGTs are responsible for catalyzing glucuronidation, a major phase II biotransformation. This reaction involves the conjugation of glucuronic acid, derived from the cofactor UDP-glucuronic acid (UDP-GlcUA), to substrates that contain suitable functional groups, most commonly hydroxyl, carboxylic acid, and amine groups [16], [17], [18]. UGTs constitute as an enzyme superfamily. The nucleotide sequences encoding 22 human UGT proteins, each approximately 530 amino acids in length, have been identified [17]. Among these, UGT1A1, 1A3, 1A4, 1A6, 1A9, 2B4, 2B7, 2B10, 2B15, and 2B17 play a major role in hepatic drug elimination [17]. Regarding UGT inhibition, studies on mitragynine and 7-hydroxymitragynine have been limited to UGT1A1 and UGT2B7. [19], [20]. Overall, 7-hydroxymitragynine showed a potential inhibitory effect on both UGT1A1 and UGT2B7 activities, with IC_50_ values of 7 µM and 26 µM, respectively [20]. In contrast, mitragynine showed less inhibition of both enzymes, with IC_50_ values exceeding 100 µM. [20]. However, the inhibitory effects of both alkaloids on other UGT isoforms remain unclear.

Using a nonselective UGT substrate, 4-methylumbelliferone (4MU), this study aimed to investigate the inhibitory effects of mitragynine and 7-hydroxymitragynine on the activities of recombinant human UGT1A1, UGT1A3, UGT1A6, UGT1A9, UGT2B7 and UGT2B15. Based on the screening results, inhibition kinetics of mitragynine and 7-hydroxymitragynine for recombinant human UGT1A3 and UGT1A9 were further performed to determine inhibition mechanism and inhibition constants (K_i_).

## 2 Materials and methods

### 2.1 Chemicals and reagents

4-Methylumbelliferone (4MU; sodium salt), 4-methylumbelliferyl-β-D-glucuronide (4MUG) and UDP-glucuronic acid (UDP-GlcUA; trisodium salt) were purchased from Sigma—Aldrich (Singapore); mitragynine (97 % purity) and 7-hydroxymitragynine (99 % purity) were purchased from CymitQuímica (Spain). Supersome™ human UDP-glucuronosyltransferases (UGTs) including UGT 1A1, 1A3, 1A6, 1A9, 2B7 and 2B15 were purchased from Corning® (USA). Solvents and other reagents were of analytical reagent grade.

### 2.2 Inhibition effect of mitragynine and 7-hydroxymitragynine by recombinant human UGT activities

The inhibitory effects of Kratom’s constituents, mitragynine and 7-hydroxymitragynine, on recombinant human UGT1A1, UGT1A3, UGT1A6, UGT1A9, UGT2B7, and UGT2B15 activities were examined using the nonselective UGT substrate, 4MU, as the probe. Incubation conditions including 4MU concentration, protein concentration and incubation time for individual UGT isoform were as indicated in previous studies with some modification [21], [22]. In brief, a 100 µl-incubation mixture contained phosphate buffer (0.1 M, pH 7.4), MgCl_2_ (4 mM), recombinant human UGT, 4MU, mitragynine or 7-hydroxymitragynine, and UDP-GlcUA (5 mM). 4MU concentrations used corresponded to the apparent K_m_ or S_50_ values of each enzyme, as described by Uchaipichat et al. [22]. The protein concentration and incubation time used for each UGT isoform were previously optimized based on the linearity of metabolite formation [22]. UGT activities were determined in duplicate in the absence (negative control) and presence of mitragynine or 7-hydroxymitragynine (1, 10, 100 and 200 µM). The stock solutions of both chemicals were prepared in methanol, ensuring that the final concentration of the solvent in the incubations was 1.0 % (v/v), which has a negligible or minor effect on most UGT activities [22]. Diclofenac (100 µM) was used as a positive control inhibitor for all inhibition screening studies. Reactions were initiated by the addition of UDP-GlcUA and incubated at 37ºC in a shaking water bath, and terminated by the addition of 1 µl of 70 % (v/v) HClO_4_. Samples were centrifuged (5000 g) at 10ºC for 10 min, and a 40 µl aliquot of the supernatant fraction was injected into the HPLC column. HPLC conditions for 4MUG quantification were as described in a previous study [21].

### 2.3 Kinetic studies for mitragynine or 7-hydroxymitragynine inhibition on recombinant human UGT activities

Mechanism and inhibition constants (K_i_) of mitragynine or 7-hydroxymitragynine on 4MU glucuronidation by recombinant human UGT1A3 and UGT1A9 were investigated by using the Dixon plot. The plots were generated using four mitragynine or 7-hydroxymitragynine concentrations at each of the three 4MU concentrations. The 4MU concentrations spanned over the previously reported K_m_ or S_50_ values for 4MU glucuronidation kinetics [22].

### 2.4 Data analysis

All data points were reported as the mean of duplicate estimates. IC_50_ and K_i_ values were estimated using Enzfitter (Biosoft, Cambridge, UK), and are reported as the parameter ± SE of the parameter estimate. Expressions for competitive, uncompetitive, noncompetitive and mixed inhibition mechanism were fitted to experimental data. Goodness of fit was assessed from comparison of the F statistic, r^2^ values, standard error of the parameter fit, and 95 % confidence intervals.

## 3 Results

### 3.1 Inhibitory effects of mitragynine and 7-hydroxymitragynine on recombinant human UGT activities

**Fig. 1:**
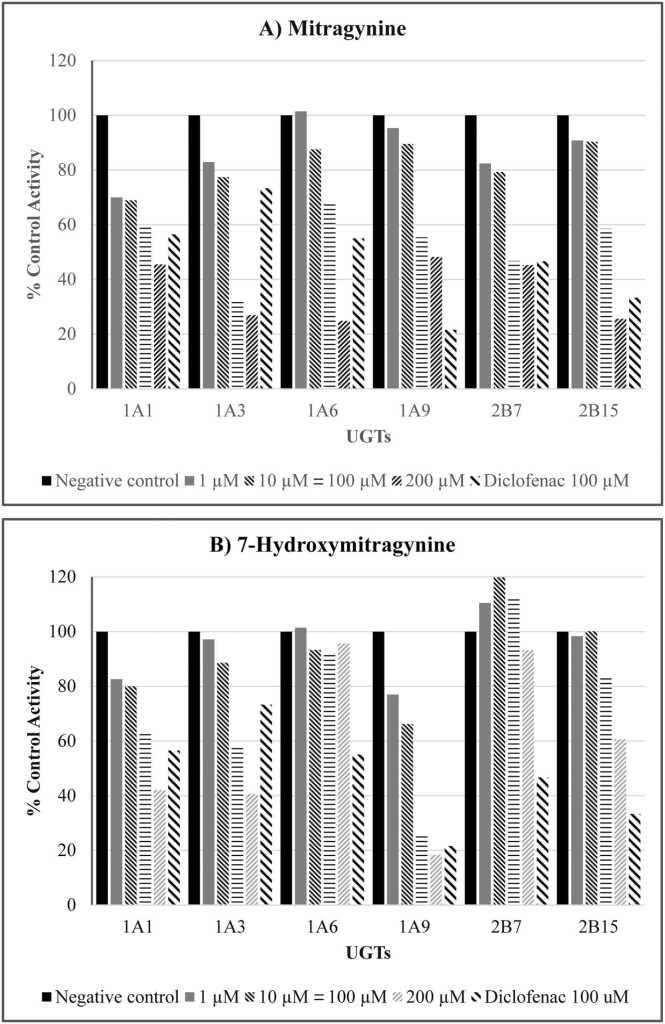
Inhibition of recombinant human UGT enzymes by mitragynine (Panel A) and 7-hydroxymitragynine (Panel B). Bars represent the mean of duplicate estimates (<10 % variance). Diclofenac (100 µM) was used as a positive control inhibitor.

Employing 4MU as a substrate, the IC_50_ values of mitragynine and 7-hydroxymitragynine on recombinant human UGT1A1, UGT1A3, UGT1A6, UGT1A9, UGT2B7 and UGT2B15 activities were evaluated. As presented in Table 1 and Fig. 1, mitragynine exhibited the highest inhibitory potency on UGT1A3 with an IC_50_ value of 72 ± 0.16 µM. Moderate inhibition potency of mitragynine was observed for UGT1A6, UGT1A9 and UGT2B15, with IC_50_ values of 121 ± 44 µM, 131 ± 11 µM, and 152 ± 24 µM, respectively, whereas the inhibition on UGT1A1 and UGT2B7 was low, with the IC_50_ values higher than 200 µM.

**Table 1:** IC_50_ values for inhibition of mitragynine or 7-hydroxymitragynine on recombinant human UGT activities.

**UGTs**	**IC**_**50**_^a^ (µM)	
	**Mitragynine**	**7-Hydroxymitragynine**
1A1	> 200	196 ± 61
1A3	72 ± 0.16	141 ± 7
1A6	121 ± 44	N/A^b^
1A9	131 ± 11	51 ± 2.4
2B7	> 200	N/A^b^
2B15	152 ± 24	> 200

a Data are presented as mean ± standard error of parameter fit

b Not available due to negligible effects observed across the concentrations screened.

The IC_50_ values and inhibitory effects of 7-hydroxymitragynine are shown in Table 1 and Fig. 1. 7-Hydroxymitragynine exhibited the highest inhibitory potency on UGT1A9, with IC_50_ value of 51 ± 2.4 µM. Moderate inhibition potency was observed for UGT1A3 and UGT1A1, with IC_50_ values of 141 ± 7 and 196 ± 61 µM, respectively. The inhibitory potency of 7-hydroxymitragynine on UGT2B15 was low, with IC_50_ values exceeding 200 µM. However, negligible effects of 7-hydroxymitragynine on UGT1A6 and UGT2B7 were observed, as the percent inhibition was less than 10 % across the concentrations screened (1–200 µM).

### 3.2 Kinetic studies for mitragynine or 7-hydroxymitragynine inhibition on 4MU glucuronidation

**Fig. 2:**
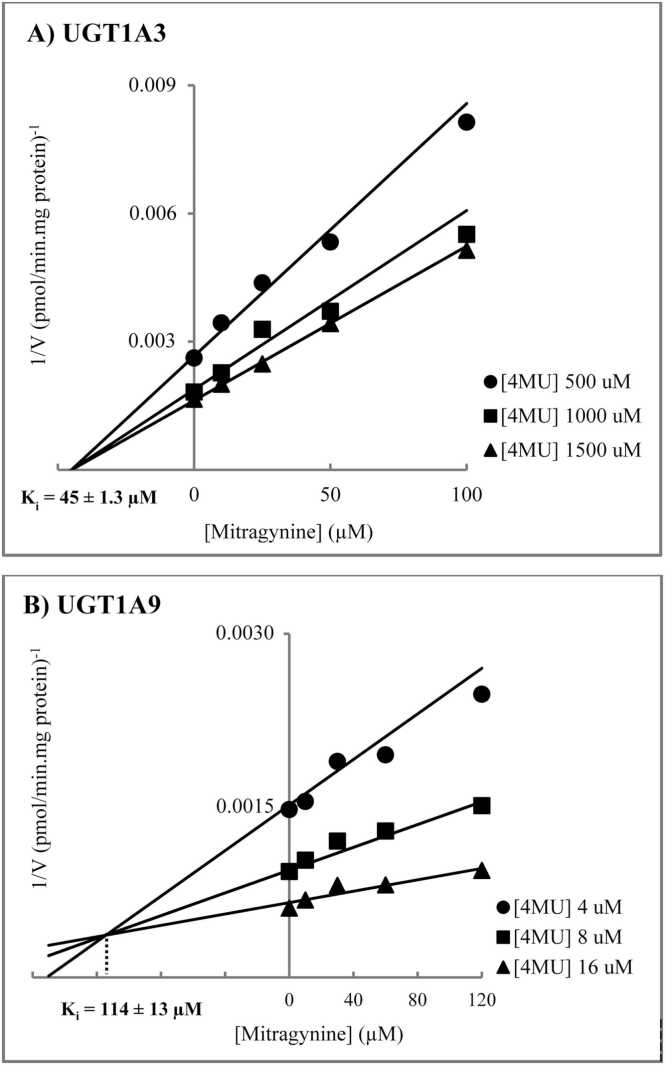
Dixon plots for the inhibition of mitragynine on 4-methylumbelliferone glucuronidation by UGT1A3 (Panel A) and UGT1A9 (Panel B).

**Fig. 3:**
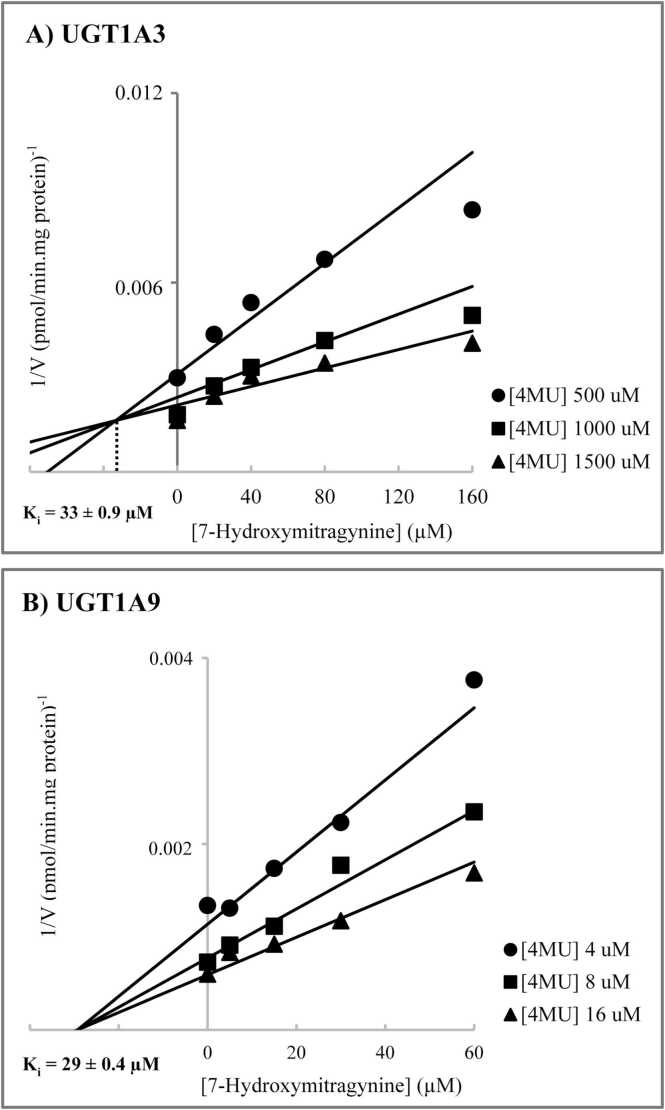
Dixon plots for the inhibition of 7-hydroxymitragynine on 4-methylumbelliferone glucuronidation by UGT1A3 (Panel A) and UGT1A9 (Panel B).

Based on the inhibition screening study, kinetic inhibition analysis was conducted for the selected UGT isoforms with potent inhibition. The inhibition mechanism and inhibition constants (K_i_) were investigated for mitragynine or 7-hydroxymitragynine inhibition on UGT1A3 and UGT1A9. As presented in Table 2 and Fig. 2, mitragynine exhibited a noncompetitive inhibition mechanism for 4MU glucuronidation by UGT1A3, with a K_i_ value of 45 ± 1.3 µM, while it competitively inhibited 4MU glucuronidation by UGT1A9, with a K_i_ value of 114 ± 13 µM. Conversely, 7-hydroxymitragynine exhibited competitive inhibition of 4MU glucuronidation by UGT1A3 and noncompetitive inhibition by UGT1A9, with K_i_ values of 33 ± 0.9 µM and 29 ± 0.4 µM, respectively (Fig. 3).

**Table 2:** Mechanism and inhibition constants for mitragynine and 7-hydroxymitragynine inhibition of 4MU glucuronidation by recombinant human UGTs.

	**Inhibition mechanism**	**K**_**i**_^a^ (µM)
**Mitragynine**		
UGT1A3	Noncompetitive	45 ± 1.3
UGT1A9	Competitive	114 ± 13
**7-Hydroxymitragynine**		
UGT1A3	Competitive	33 ± 0.9
UGT1A9	Noncompetitive	29 ± 0.4

a Data are presented as mean ± standard error of parameter fit

## 4 Discussion

As Kratom use increases, growing concerns have emerged regarding its potential for herb-drug interactions with liver enzymes, especially UDP-glucuronosyltransferases (UGTs). While Kratom's inhibitory effects on cytochrome P450 enzymes are well-documented, evidence regarding its impact on UGT-mediated glucuronidation, a key phase II drug elimination process, remains limited. This study explored the inhibitory effects of Kratom constituents, mitragynine and 7-hydroxymitragynine, on 4MU glucuronidation by a panel of recombinant human UGTs, including UGT1A1, UGT1A3, UGT1A6, UGT1A9, UGT2B7, and UGT2B15. Diclofenac (100 µM) was employed as a positive control inhibitor, and its inhibition potency on UGTs (see Fig. 1) was consistent with findings from a previous study [22]. Among the six UGT isoforms screened, the inhibitory potency of mitragynine and 7-hydroxymitragynine varied. A potential inhibitory effect (IC_50_ <100 µM) was observed for mitragynine on UGT1A3 and 7-hydroxymitragynine on UGT1A9. Regarding UGT1A1 and UGT2B7, enzymes that play an important role in drug elimination in the liver, the overall inhibition exhibited low to moderate potency, with negligible effects observed for 7-hydroxymitragynine and UGT2B7.

Among the UGTs investigated in the screening study, UGT1A1, UGT1A9, and UGT2B7 are likely the most important enzymes involved in drug metabolism in the liver [17], [23], [24]. Although mitragynine exhibited the highest inhibition potency on UGT1A3 with IC_50_ values of 72 ± 0.2 µM, its inhibition was moderate for UGT1A9 (IC_50_ >100 µM) and low for UGT1A1 and UGT2B7 (IC_50_ >200 µM). The IC_50_ values for mitragynine's inhibition of UGT1A1 and UGT2B7 are consistent with previous reports [20]. However, the results for UGT2B7 differ from those of a study using human liver microsomes as the enzyme source, which reported strong inhibition potency (IC_50_ = 8.11 µM) [19].

Regarding 7-hydroxymitragynine, the highest inhibitory potency was observed for UGT1A9, with an IC_50_ value of 51 ± 2.4 µM, while moderate potency was found for UGT1A1 (IC_50_ 196 µM), and negligible effects were observed for UGT2B7. These results for UGT1A1 and UGT2B7 are inconsistent with those reported previously [20]. Haron et al. found that 7-hydroxymitragynine exhibited potent inhibition of UGT1A1 and UGT2B7, with respective IC_50_ values of 7.13 µM and 26.44 µM. The variability in IC_50_ values between studies could be attributed to several factors, including differences in enzyme sources, incubation conditions, and substrates. Notably, the recombinant protein concentration used in the study by Haron et al. was approximately three to five times lower than that used in the present study and previous report [22]. Additionally, the salted form of 4MU employed may affect its solubility during incubation. While 4MU sodium salt was used as the substrate in this study, the specific form of 4MU was not specified in the study by Haron et al. [20], presumably that the unsalted compound may have been employed.

Based on the inhibitory potential observed in the screening study, kinetic studies of mitragynine and 7-hydroxymitragynine inhibition on 4MU glucuronidation were further evaluated for recombinant UGT1A3 and UGT1A9. Notably, UGT1A9 is one of the enzyme that play a major role for drug elimination in the liver [17], [23], [24]. A competitive inhibition mechanism was observed for mitragynine inhibition of UGT1A9 and 7-hydroxymitragynine inhibition of UGT1A3, suggesting that both alkaloids could potentially be glucuronidated by these specific UGTs. However, this remains inconclusive, as no evidence of such glucuronidated metabolites has been observed in humans [25], [26]. In addition, mitragynine is known to be extensively metabolized through oxidative processes [25], [26], [27].

The K_i_ values for mitragynine and 7-hydroxymitragynine inhibition of UGT1A3 and UGT1A9 were 45 ± 1.3 µM and 114 ± 13 µM, and 33 ± 0.9 µM and 29 ± 0.4 µM, respectively. Pharmacokinetic studies in humans have reported that the maximum plasma concentrations (C_max_) of mitragynine was 0.081 µM after a single oral dose [27] and 0.263 µM after multiple oral doses of Kratom tea [28], whereas the C_max_ of 7-hydroxymitragynine was 0.016 µM after a single dose [27]. Based on the basic static model prediction [23], [29], the potential for herb-drug interactions arising from glucuronidation inhibition by mitragynine and 7-hydroxymitragynine is unlikely, as the *in vitro* K_i_ values are relatively high compared to the C_max_ values of both alkaloids observed in humans. However, due to the complexity of extrapolating *in vitro* results to *in vivo* scenarios, such as the identification of true K_i_ values and variability in Kratom dosing that may affect the C_max_ values, the potential for interaction cannot be entirely ruled out. In addition, future studies on the effects of other alkaloids in Kratom leaves and the potential inhibition of other UGT isoforms should be warranted.

In conclusion, Kraton constituents, mitragynine and 7-hydroxymitragynine, inhibited the activities of UGT1A1, UGT1A3, UGT1A6, UGT1A9, UGT2B7 and UGT2B15 on 4-MU glucuronidation to varying degrees. The highest potency was observed for mitragynine inhibition of UGT1A3 and 7-hydroxymitragynine inhibition of UGT1A9. Although the prediction screening indicated an unlikely potential for herb-drug interaction in humans, further investigation is warranted to comprehensively assess this potential.

## CRediT authorship contribution statement

**Verawan Uchaipichat:** Writing – original draft, Methodology, Investigation, Funding acquisition, Formal analysis, Data curation, Conceptualization.

## Declaration of Competing Interest

The authors declare that they have no known competing financial interests or personal relationships that could have appeared to influence the work reported in this paper.

## Data Availability

No data was used for the research described in the article.
